# On the Transformation of the Two Aortæ into One in Embryonic Vertebrata

**Published:** 1852-05

**Authors:** 


*On the Transformation of the two Aortæ into one in Embryonic Vertebrata.*—M. Serres read a paper before the Academy of Sciences of Paris, on the 22nd of December, wherein he confirms the experiments of Dr. Allen Thompson and Mr. Milne Edwards, touching the time of embryonic life, when the originally double aorta is conjoined into one. M. Serres placed the embryo chicken with the omphalo-mesenteric membrane on a plate of glass; as the action of the air and cold arrests the circulation, the blood becomes coagulated in the vessels, the transparency of which allows the sanguineous injection to be seen. By means of a serie; of preparations thus arranged, the above mentioned union is seen to take place towards the middle of the dorsal region from the fiftieth to the sixtieth hour; it then extends upwards from the sixty-fifth to the seventieth hour, and progesses downwards from this latter period. Thus, at the end of the third day, and, at the latest, of the eighty-fifth hour, the two arterial trunks are united, and form but one vessel.—*London Lancet.*

